# Minimally invasive distal pancreatectomy for PNETs: laparoscopic or robotic approach?

**DOI:** 10.18632/oncotarget.17513

**Published:** 2017-04-28

**Authors:** Jiaqiang Zhang, Jiabin Jin, Shi Chen, Jiangning Gu, Yi Zhu, Kai Qin, Qian Zhan, Dongfeng Cheng, Hao Chen, Xiaxing Deng, Baiyong Shen, Chenghong Peng

**Affiliations:** ^1^ Department of Pancreatic Surgery, Ruijin Hospital, Shanghai Jiao Tong University School of Medicine, Shanghai, P.R. China; ^2^ Research Institute of Digestive Surgery, Ruijin Hospital, Shanghai Jiao Tong University School of Medicine, Shanghai, P.R. China

**Keywords:** PNETs, distal pancreatectomy, robotic surgery, laparoscopic surgery

## Abstract

**Background:**

The most effective and radical treatment for pancreatic neuroendocrine tumors (PNETs) is surgical resection. Minimally invasive surgery has been increasingly used in pancreatectomy. Initial results in robotic distal pancreatectomy (RDP) have been encouraging. Nonetheless, data comparing outcomes of RDP with those of laparoscopic distal pancreatectomy (LDP) in treating PNETs are rare. The aim of this study was to compare the safety and efficacy of RDP and LDP for PNETs.

**Methods:**

From September 2010 to January 2017, operative parameters and perioperative outcomes in an initial experience with 43 consecutive patients undergoing RDP were collected and compared with those in 31 patients undergoing LDP.

**Results:**

Patients undergoing RDP and LDP demonstrated equivalent age, sex, ASA score, tumor location and tumor size. Operating time, length of resected pancreas, postoperative length of hospital stay and rates of conversion to open, pancreatic fistula, transfusion and reoperation were not statistically different. Patients in the RDP group were associated with significantly higher overall (79.1 *vs*. 48.4 %, *P* = 0.006) and Kimura spleen preservation rates (72.1 *vs*. 16.1%, *P* < 0.001) and had reduced risk of excessive blood loss (50 *vs*. 200mL, *P* < 0.001). Oncological outcomes in this series were superior for the RDP group with more lymph node harvest for G2 and G3 PNETs (3.5 *vs*. 2, *P* = 0.034).

**Conclusions:**

Both RDP and LDP are efficacious and safe methods in treating PNETs located in the body or tail of pancreas. Robotic approach offers advantages with less intraoperative blood loss, higher spleen preservation rate and more lymph node harvest. It may be sensible to choose RDP for patients who fit indications for scheduled spleen preservation.

## INTRODUCTION

Pancreatic neuroendocrine tumors (PNETs), as the name implies, are a rare group of neoplasms that originate from the endocrine portion of the pancreas, which have an incidence of 2-3 per 100,000 individuals per year, and constitute only about 1% to 2% of all pancreatic neoplasms [1–2]. A high proportion of PNETs are characterized as sporadic and lack germline mutations [3]. PNETs more frequently arise in patients between the age of 40 and 60, though they can develop at any age. They could be divided depending on the secreted hormones resulting different symptoms. Non-functioning (NF) tumors are the most common with up to 60~90% of PNETs, whereas functioning PNETs like gastrinomas, insulinomas and others develop in about 30, 10 and < 5% of patients [4–6].

Surgical resection, premised on definite diagnosis and accurate location of tumor, is considered to be a radical and reliable treatment for primary PNETs, because it is associated with increased survival [7]. With improvements in science and technology, the use of minimally invasive surgery has been furthered. In 1996, Cuschieri performed the world’s first laparoscopic distal pancreatectomy (LDP) [8]. In the same year, Gagner [9] introduced an initial experience of laparoscopic surgery for islet cell tumor. From then on, many reports have confirmed that laparoscopic method for PNETs is safe and feasible, though the majority of them are summarized from limited experience without long-term follow-up [10–14]. LDP is the most commonly performed and the most mature laparoscopic pancreatic surgical procedure [16], as it does not contain anastomoses or complex reconstruction of alimentary tract compared to the highly challenging laparoscopic pancreaticoduodenectomy. Minimally invasive surgery might be a promising treatment for insulinoma or NF-PNETs, with concrete proof in the literature supporting better outcomes of the laparoscopic approach compared with open surgery [15–17]. Recently developed robotic surgical system has overcome the limitations of laparoscopic technology by providing an isometric 3D view and a high level of flexibility for manipulation. The first case of robotic distal pancreatectomy (RDP) was reported in 2002 [18], ushering in a new era in minimally invasive pancreatic surgery. In 2003, Melvin [19] reported the first known case in which da Vinci robotic surgical system was used for resecting PNET. Also, RDP is also believed to be helpful to increasing spleen-preservation rate, due to its inherent advantages [20–27]. Our hypothesis is that, as compared with LDP, high dexterity and clear vision of RDP would produce uniformly superior results in treating PNETs. Therefore, a retrospective analysis was performed.

## MATERIALS AND METHODS

### Design and study population

The study population comprised a cohort of 74 consecutive patients with PNETs who underwent minimally invasive approach of distal pancreatectomy at the Department of Hepato-bilio-pancreatic Surgery, Ruijin Hospital affiliated to Shanghai Jiaotong University School of Medicine, a multidisciplinary, academic tertiary care facility and the largest pancreatic surgery center in mainland China with an annual case volume of more than 500 pancreatic surgeries between September 2010 to January 2017. Laparoscopic approach has been our choice for distal pancreatectomy for more than a decade, meanwhile we started to perform pancreatic surgeries using the daVinci^®^ system (Intuitive surgical Inc. Sunnivale, CA, USA) in March 2010. Demographic data were collected on each patient: gender, age, body mass index (BMI) at the time of the operation, symptoms, and American Society of Anesthesiologists (ASA) physical status. Clinical and pathological variables mainly included operative time, estimated intraoperative blood loss, transfusion rate, conversion rate, length of postoperative hospital stay (PHS), R_0_ resection rate, tumor histology, tumor size, postoperative pancreatic fistula or other complications, mortality and follow-up. All clinical data obtained in this study were retrospectively analyzed in a prospectively maintained database. All demographic and perioperative data were documented using a computerized hospital information system database. The design of this study was approved by the institutional review board at Ruijin hospital in accordance with the latest version of the Declaration of Helsinki. The inclusion criteria were: solitary insulinoma or NF-NET, located in the body / tail of the pancreas, with no radiologic evidence of high-grade malignancy and could not be treated with enucleation. All included patients during this same period had to be eligible for both RDP and LDP to minimize possible selection bias. All patients included were well informed of the advantages and disadvantages of RDP and LDP by independent research nurses. The choice of either approach was at the sole discretion of the patient. Patients were excluded if they had presence of serious cardiopulmonary dysfunction or hepatorenal insufficiency, had a previous history of upper abdominal surgery, had lesions that were deep-seated or in its late stages (e.g., due to the involvement of the blood vessels or diffuse liver metastases) or underwent an operative treatment for gastrinoma (these patients were not considered candidates for a minimally invasive approach according to recent guidelines) [28–30]. There was no restriction on age [31]. The indications for scheduled splenic preservation in our center were: a non-malignant pancreatic tumor or a suspected malignant pancreatic tumor smaller than 2 cm (AJCC stage IA) and without significant compression or involvement of the splenic vessel shown on preoperative CT scan. The analysis of outcomes was performed on an intent-to-preserve basis, with the outcomes of splenectomy analyzed as consequences of the intended splenic preservation procedure.

### Main outcome measures

Pancreatic fistula was defined according to the guidelines of the International Study Group on Pancreatic Fistula (ISGPF) [32]. Post-pancreatectomy haemorrhage was defined according to the guidelines of the International Study Group of Pancreatic Surgery [33]. Operative time was calculated as the time between skin incision and skin closure of the last port. Postoperative morbidities were evaluated according to the Clavien–Dindo classification system, and a major complication was defined as Clavien–Dindo classification≥3 [34]. Mortality was defined as death within the 60 days after surgery in or out of hospital. Out patient records combined with telephone interviews were used for follow-up. The follow-up period was defined as the period between the day of operation and the day of the last follow-up. Follow-up was updated in January 2017.

### Surgical methods

**Figure 1:**
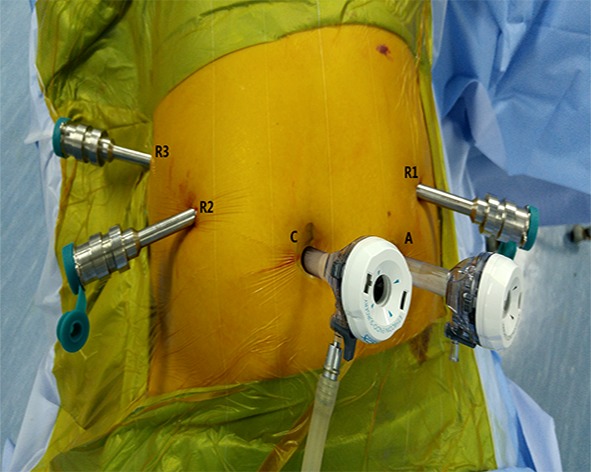
Trocar port placement in robotic distal pancreatectomy C: Camera port (12 mm); R1: No. 1 main operating arm port (8 mm); R2: No. 2 main operating arm port (8 mm); A: Assistant operating port (12 mm); R3: No. 3 auxiliary arm port (8 mm).

**Figure 2:**
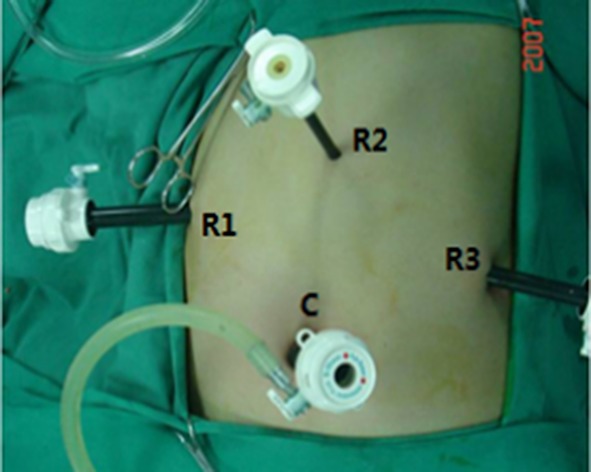
Location of trocar ports during laparoscopic distal pancreatectomy C: Laparoscopic port (12 mm); R1: No. 1 operating port (12 mm); R2: No. 2 operating port (8 mm); R3: No. 3 operating port (12 mm).

**Figure 3:**
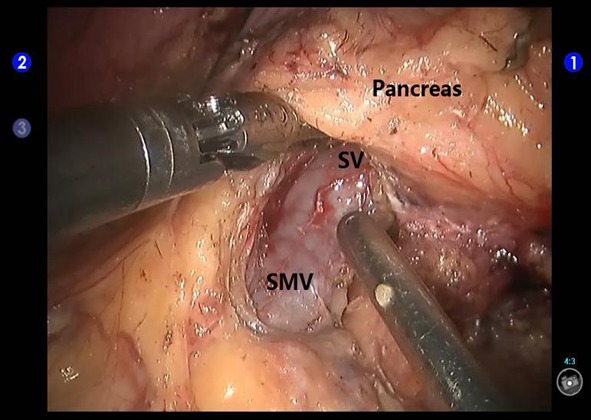
Creation of a retropancreatic tunnel during robotic distal pancreatectomy SV: the splenic vein; SMV: the superior mesenteric vein.

**Figure 4:**
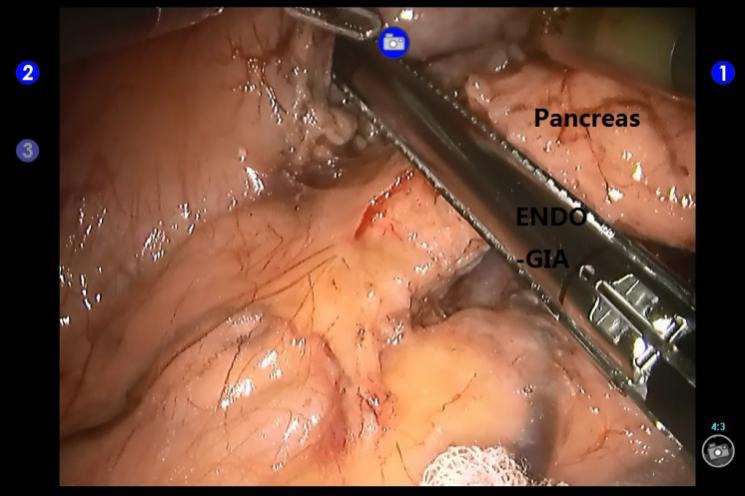
Transection of the pancreas using an Endo-GIA stapler during robotic distal pancreatectomy

**Figure 5:**
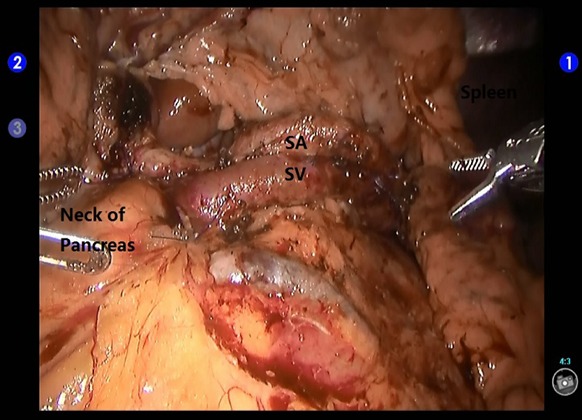
Exposure of the splenic artery and vein during spleen-preserving robotic distal pancreatectomy. SA: the splenic artery; SV: the splenic vein.

The patients received routine general anesthesia and were placed in the supine position with their legs apart and head raised (the right side was raised to a 30° angle). Pneumoperitoneum was achieved by puncturing the tissue surrounding the umbilicus. In the robotic surgery group, the trocars were positioned according to the 5-hole method [35] (Figure 1), then the da Vinci^®^ surgical arm cart was docked, and each manipulator was installed. Similarly, four trocars were placed in the conventional laparoscopic surgery group (Figure 2). The procedure of LDP and RDP is basically same. The classical technique was used for splenectomy: The gastrocolic ligament was incised so that it would be possible to enter the lesser sac and to examine the infiltration of the tumor. The splenic artery was dissected from the superior margin of the pancreatic body and ligated using vascular clips. A retropancreatic tunnel was created (Figure 3) and the pancreatic neck was mobilized at a distance of approximately 1 cm from the mass using Endo-GIA cutting stapler (60 mm, 2.5) (Tyco Inc., U.S.A.) (Figure 4), and the splenic veins were transected. Cut off the short gastric vessels, then the pancreatic body and tail containing the tumor and the spleen were completely removed. Two techniques were used for preserving spleen: The Kimura technique with isolation but not cut off the splenic vessels was preferred [36], and the Warshaw technique without preservation of the splenic vessels but with preservation of the short gastric vessels and left gastro-omental vessels was attempted if the Kimura technique failed [37] (Figure 5). One peritoneal drain was placed at the remnant of the pancreas, and an additional drain was placed in the splenic fossa for patients undergoing splenectomy.

### Statistical analysis

Statistical analysis was performed using SPSS Version 19.0 for Windows (SPSS, Inc., Chicago, IL, USA). Normality was measured using the Shapiro-Wilk test. Normally distributed variables were expressed as mean ± standard deviation (SD) and compared using the two independent-samples Student’s *t* test. Non-normal variables were reported as medians with interquartile range (IQR), and the Man-Whitney *U* test was used to test the difference. Categorical data were expressed as *n* (%) and compared using the Chi-square test or Fisher’s exact test. A two-sided *P* value < 0.05 was considered statistically significant.

## RESULTS

Altogether, 74 patients with PNETs underwent minimally invasive distal pancreatectomy (RDP group, *n*=43; LDP group, *n*=31). Some of them also underwent splenectomy. As shown in Table 1, patients’ characteristics and pathologic details were summarized. There were no significant differences between groups in terms of demographic data and perioperative physical status, including gender, age, BMI and ASA scores. Prior to surgery, 17 patients were found to have pancreatic space-occupying lesions, 24 patients experienced hypoglycemia symptoms, and 2 patient experienced upper abdominal pain and discomfort in RDP group. The parallel data in LDP group is 9, 19, 3, respectively. Blood sugar of all the patients with insulinoma returned to a normal level after operation. Most PNETs were grade G1 in each group. There were respectively 8 and 7 patients in RDP and LDP groups who had G2 or G3 tumors (Table 1).

**Table 1:** Characteristics and pathologic data of patients with PNETs undergoing RDP and LDP

Variables	RDP (*n* = 43)	LDP (*n* = 31)	*P* value
Gender [*n* (%)]			
Male	20 (46.5)	12 (38.7)	0.504
Female	23 (53.5)	19 (61.3)	0.504
Age [mean±SD (years)]	47.9±10.5	48.7±12.3	0.766
Symptons [*n* (%)]	26 (60.5)	22 (70.1)	0.350
Hypoglycemia	24 (55.8)	19 (58.8)	0.638
Abdominal discomfort	2 (4.7)	3 (11.3)	0.644
BMI [mean±SD (kg/m2)]	23.9±3.2	23.3±2.7	0.401
ASA class [*n* (%)]			
I	32 (74.4)	22 (70.1)	0.742
II	11 (25.6)	9 (29.0)	0.742
III	0	0	1.000
Type of pathology [*n* (%)]			
Insulinoma	24 (55.8)	19 (61.3)	0.638
G1^a^	22 (51.2)	17 (54.8)	0.755
G2^b^	2 (4.7)	1 (3.2)	1.000
G3^c^	0 (0.0)	1 (3.2)	1.000
Non-functional neuroendocrine tumor	19 (44.2)	12 (38.7)	0.638
G1	13 (30.2)	7 (22.6)	0.465
G2	5 (11.6)	4 (12.9)	1.000
G3	1 (2.3)	1 (3.2)	1.000

*RDP* robotic distal pancreatectomy, *LDP* laparoscopic distal pancreatectomy, *BMI* body mass index, *ASA* American Society of Anesthesiologists

^a^ Ki67 index ≤ 2

^b^ Ki67 index 2-20

^c^ Ki67 index >20

All patients underwent an R_0_ resection. Median tumor size in RDP group was 1.6 cm (IQR 1.3-2.5 cm) and in LDP group was 1.6 cm (IQR 1.2-2.2 cm) (*P* = 0.720). The blood loss during operation of RDP group (50mL, IQR 50-100mL) was obviously lower than that of LDP group (200mL, IQR 20-900mL; *P* < 0.001). This result suggests an improvement in major hemorrhage prevention. Furthermore, the total spleen-preservation rate in RDP group (79.1%) is higher than that in LDP group (48.4%) significantly (*P* = 0.006), among them Kimura technique had a higher usage in RDP group (72.1% *vs*. 16.1%; *P* < 0.001). No distinct difference was found in tumor location (*P* = 0.291), operating time (139.3 min *vs*.133.4 min; *P* = 0.625), transfusion rate (9.3% *vs*. 12.9%; *P* = 0.713), tumor size (1.6cm *vs*. 1.6cm; *P* = 0.720), rate of convertion to open (0 *vs*. 0) and hospital stay after surgery (12.8 days *vs*.14.4 days; *P* = 0.327). Complications are shown in Table 2. Two approaches had similar incidences of pancreatic fistula after operation (25.6% *vs*. 38.7%; *P* = 0.229). In RDP group, 7 cases were classified to have ISGPF grade A, 4 was classified to have ISGPF grade B, while in the LDP group, the number of Grade A, B, C was 7, 4, 1, respectively. One case of wound infection was observed in LDP group. The patient with postoperative hemorrhage caused by pancreatic fistula (Grade C) in LDP group underwent an emergent exploratory laparotomy to staunch bleeding, and an active bleeder was identified at a branch of splenic artery. Neither group had perioperative death. The median follow-up periods were 16 months (IQR 1-75 months) for RDP group and 23 months (range 9-72 months) for LDP group, respectively. Over the follow-up period there were 12 (33.3%) cases of new PNETs in the residual pancreas, 7 (36.8%) in RDP group and 5 (29.4%) in LDP group. Three patients developed new PNETs 1-2 cm were suggested to receive the secondary surgery to enucleat tumor. Among them, one was in LDP group (5.9%) and the other two were in RDP group (10.6%). No patients developed a diabetes mellitus after operation. Besides, no organic hyperinsulinism was observed in the recovery period (Table 2).

**Table 2:** Operative and postoperative data following laparoscopic and robotic distal pancreatectomy

Variables	RDP	LDP	*P* value
Number of patients (*n*)	43	31	NA
Operation time [mean±SD (min)]	139.3±56.9	133.4±41.8	0.625
Blood loss [median (IQR), mL]	50 (50-100)	200 (160-300)	< 0.001
Transfusion [*n* (%)]	4 (9.3)	4 (12.9)	0.713
Tumor size [median (IQR), cm]	1.6 (1.3-2.5)	1.6 (1.2-2.2)	0.720
Tumor location [*n* (%)]			
Body	13 (30.2)	6 (19.4)	0.291
Tail	30 (69.8)	25 (80.6)	0.291
Spleen preservation [*n* (%)]	34 (79.1)	15 (48.4)	0.006
Warshaw technique	3 (7.0)	10 (32.3)	0.011
Kimura technique	31(72.1)	5 (16.1)	< 0.001
Conversion to open [*n* (%)]	0 (0.0)	0 (0.0)	1.000
R_0_ resection [*n* (%)]	43 (100)	31 (100)	1.000
Length of resected pancreas [mean±SD (cm)]	6.2±1.3	6.4±1.4	0.438
PHS [mean±SD (day)]	12.8±6.8	14.4±7.2	0.327
Complication [*n* (%)]	11 (25.6)	13 (41.9)	0.138
POPF	11(25.6)	12 (38.7)	0.229
Grade A	7 (16.3)	7 (22.6)	0.495
Grade B	4 (9.3)	4 (12.9)	0.713
Grade C	0 (0.0)	1 (3.2)	0.419
Wound infection	0 (0.0)	1 (3.2)	0.419
Hemorrhage	0 (0.0)	1 (3.2)	0.419
Reoperation because of complication [*n* (%)]	0 (0.0)	1 (3.2)	0.419
Perioperative mortality [*n* (%)]	0 (0.0)	0 (0.0)	NA
Postoperative follow-up [median (range), month]	16 (1-75)	23 (9-72)	0.056
New pNET during follow-up [*n* (%)]	7 (16.3)	5 (16.1)	0.986
Reoperation because of new pNETs [*n* (%)]	2 (4.7)	1 (3.2)	1.000

Bold values indicate statistical significance

*RDP* robotic distal pancreatectomy, *LDP* laparoscopic distal pancreatectomy, *PHS* postoperative hospital stay, *POPF* postoperative pancreatic fistula, *NA* not applicable

The pathological outcomes for patients undergoing RDP and LDP for G2 or G3 tumors are listed in Table 3. More lymph nodes were resected in RDP group (3.5, IQR 3-7.8 *vs*. 2, IQR 1-2; *P* = 0.034). Patients in two groups had a similar tumor size, R_0_ resection rate and positive nodes.

**Table 3:** Pathological outcomes following distal pancreatectomy for G2 or G3 PNETs

Variables	RDP	LDP	*P* value
Frequency (*n*)	8	7	0.675
Tumor size [median (IQR), cm]	2.5 (1.8-3.0)	2.0 (1.6-4.0)	0.727
R_0_ resection [*n* (%)]	8 (100)	7 (100)	1.000
Nodal harvest [median (IQR)]	3.5 (3-7.8)	2 (1-2)	**0.034**
Positive lymph nodes [*n* (%)]	10 (27.0)	3 (21.4)	0.682

Bold values indicate statistical significance

*RDP* robotic distal pancreatectomy, *LDP* laparoscopic distal pancreatectomy

## DISCUSSION

Though there are disputes about the operative approach to PNETs, minimally invasive technology has been used more and more in pancreatic resection in order to lessen the surgical traumas and complications that come with open surgery [12, 38–39], particularly for distal pancreatectomy (DP), the most commonly used surgical procedures completed by the laparoscopic method at present, since the process is relatively straightforward without reconstruction of alimentary tract and can be easily performed within a short time [40]. Recent data indicate that LDP is beneficial and can be safely used to treat NETs located in the body or tail [16,41]. DaVinci robot system,, made by Intuitive Surgical, offers a new option for minimally invasive pancreatic surgery. As compared to conventional laparoscopy, surgical robot system provides surgeons with enhanced visual control and operation flexibility. However, there is not any report about which approach is more outstanding for DP in treating PNETs. Therefore, we compared retrospective outcomes of robot-assisted and laparoscopic DP in patients with insulinoma or non-functional neuroendocrine tumor, two of the most common types of PNETs.

Comparion of minimally invasive approaches for DP have been made in the following four studies so far. Daouadi *et al*. [25] retrospectively analyzed the clinical data of 124 patients between 2004 and 2011 and found that RDP had a lower rate of conversion to open (0% in RDP *vs*. 16 % in LDP; *P* < 0.05), lower intraoperative blood loss (375mL, range 300-550 in RDP *vs*. 550mL, range 400-650 in LDP; *P* < 0.05), and shorter operating time (293±93min in RDP *vs*. 372±141min in LDP; *P* < 0.01), respectively. Chen *et al*. [27] evaluated 80 distal pancreatectomy cases scheduled for Spleen preservation (SP) and 39 cases for splenectomy. They found that RDP was beneficial for the spleen-preserving patients in the following aspects: blood loss (median 100 mL in RDP group *vs*. 300 mL in LDP group; *P* < 0.001), transfusion frequency (2.1% in RDP group *vs*. 18.2 % in LDP group; *P*=0.036), OT (median 120 min in RDP group *vs*. 200 min in LDP group; *P* < 0.001), overall SP rates (95.7% in RDP group *vs*. 39.4 % in LDP group; *P* < 0.001), Kimura SP rates (72.3% in RDP group *vs*. 21.2 % in LDP group; *P* < 0.001) and mean PHS (10.2 days in RDP group *vs*. 14.5 days in LDP group; *P*=0.019). While among matched patients scheduled for splenectomy, RDP had no advantages over LDP in terms of intraoperative and postoperative outcomes. Another work [20] claimed that RDP operation required a longer time than LDP (221.4 min *vs*. 173.6 min; *P*=0.026), but there were no marked differences in amount of blood loss, spleen-preservation rate, post-operative hospital stay and overall morbidity rate between two groups. Ryan and colleagues [42] reported similar results in a prospective observational study.

In recent years, more and more surgeons suggest preserving the spleen during DP in patients with benign tumors or low-grade malignant tumors. Spleen-preserving DP (SPDP) can be performed in two manners, with the removal or with the preservation of the splenic vessels. The former procedure was initially reported by Warshaw [37] in 1988, and the latter was first reported by Kimura [36] in 1996. Schwarz *et al*. [43] carried out a retrospective study on 326 patients who underwent DP and concluded that in contrast with the spleen-removing group, the median survival period was significantly longer in the spleen-preserving group, even though splenectomy did not affect postoperative recovery. Therefore, we recommended that combined splenectomy be avoided during DP in patients with PNETs, if the splenic vessels are not invaded. There are many small vessels connecting pancreatic body and tail, and splenic artery and vein (usually 5-7 branches) have short vascular pedicles, the vascular walls are easily ruptured during dissection and result in bleeding, thus the key point of SP-LDP is to safely separate pancreatic body and tail from splenic vessels [35]. Robot-assisted laparoscopic systems can effectively improve the accuracy of the surgical procedure, especially in the process of dissecting the splenic vessels and creating retropancreatic tunnel, improving the safety of surgery and the spleen-preserving rate. Our present results fully demonstrate that robotic approach was more beneficial for patients with PNETs undergoing DP. Besides, this might additionally benefits patients, as the occurrence of some complications following the Warshaw technique, for instance, secondary infection and postoperative spleen infarction, could be avoided by using Kimura technique [44–48]. It is the superior technical characteristics of the robotic surgical system (such as the augmented, high-quality, three-dimensional vision and the precise endowrist instrument motion) that ensure the feasibility and safety of distal pancreatectomy with spleen and the splenic vessels preservation.

As minimal invasive techniques has been widely employed in the treatment of malignant diseases, the primary concern is oncological safety. Some previous researches showed that lymph node metastasis of PNETs indicates the patient will have a poor prognosis [49–51] and it positively correlated with pathological grading (15-20% in G1, 30-40% in G2, and > 50% in G3) [52]. However, those studies did not clearly show that omitting lymphadenectomy would increase the rate of local recurrence, which is a requirement for recommending lymph node dissection in PNETs. Meanwhile, some other reports advocated whether regional lymph node metastasis of PNETs could produce oncologic effects need to be questioned. Tsutsumi *et al*. [53] failed to prove lymph node metastasis is independently associated with tumor recurrence in their multivariate analysis. Brinbaum [54], Gratian [55] and Bilimoria *et al*. [56] also reported similar results. A retrospective study of patients with NF-NET-G1 who underwent limited pancreatectomy or distal pancreatosplenectomy showed no adverse impact in oncologic outcomes and suggested that local lymph nodes resection should not be recommended as routine procedure [57]. Indeed, most well differentiated insulinomas and NF-NETs located in distal pancreas are quite small and lymph node metastasis is seldom noted, in particular, with the lack of radiographic evidence of lymph node inolement [58–59]. Based on our experience and before-mentioned theories, we adopted simple segmental pancreatectomy in well-selected cases that predicted before operation by radiologic techniques. When we counted the lymph nodes acquired from G2 and G3 PNETs, it was encouraging to found evidence of the superiority of RDP. Other oncological outcomes were similar in both groups. 43 patients with an insulinoma became symptom-free and were thought to be biochemically cured. No one died from their tumors, which executives attribute to we carried out active surgical treatment at a relatively small tumor size.

Patients with PNETs have a high recurrence rate, especially people who have a strong genetic predisposition [60]. In our study, over the follow-up period, 12 of the 74 patients (16.2 %) developed new PNETs, and three (4.1 %) got a second operation. Rate of second operation were the same in both groups (2/43, 4.7 % in RDP group vs. 1/31, 3.2 % in LDP group). The majority of patients with PNETs are young or middle-aged, some of them may need more than one pancreatic surgery. Accordingly and in view of the interests of the patients, a robot-assisted precision pancreatic resection can be a perfect mean through which they can improve the quality of their life by reducing some chronic diseases including diabetes mellitus, digestive disorders, and portal vein thrombosis after splenectomy.

Robotic technology truly give birth to corresponding materials costs [35, 61–62], so it is easy to understand that the pantients who chose robotic approach need to pay a higher initial fee. This health economic issue could be examined and discussed in our subsequent studies. Before establishing this kind of research, it is important for us to be clear on how to reflect and eliminate the effects of inflation. Despite all this, it is worth performing RDP for patients who fit indications for scheduled spleen preservation, in order to raise the probability.

Though the present study is the first retrospective comparison between RDP and LDP in treating PNETs for short-term outcomes, a few limitations still exist. First of all, the retrospective study at a single center may make the outcomes subject to a general selection bias. We tried to minimize it by retrieving all data from a prospective database. And, Secondly, due to the small sample, it would be hard to draw any strong conclusions. Nevertheless, we should mention at this point that patients with PNETs who satisfy the indications for surgery are uncommon. Thirdly, during the study period, the operative indication for non-functional PNETs has been changed. Fourthly, there were limited cases with malignant pathologies (G3) in this study, it need to get more cases and conduct prospective study in the future.

## CONCLUSIONS

The present study suggests that both RDP and LDP are valid methods of the minimal invasive treatment for NETs located in distal pancreas. Robotic approach potentially offers advantages with less blood loss in operation and higher spleen preservation rate. It may be wise to choose RDP for patients who fit indications of scheduled spleen preservation. Larger series, prospective studies and randomized clinical trials comparing outcomes of robotic and laparoscopic DP are needed to validate the above-mentioned potential advantages and determine the clinical position of each approach.
